# Adaptative Strategies in *Gymnocalycium* Species (Cactaceae) and the Presence of Ectomycorrhizae Associated with Survival in Arid Environments

**DOI:** 10.3390/plants12152774

**Published:** 2023-07-26

**Authors:** María E. Soto Acosta, Mario Perea, Ana I. Ruiz, Mirna Hilal, Patricia L. Albornoz, María I. Isla

**Affiliations:** 1 CEVIR and Cátedra de Fisiología Vegetal, Facultad de Ciencias Exactas y Naturales, Universidad Nacional de Catamarca, Av. Belgrano 300, San Fernando del Valle de Catamarca K4700CTK, Catamarca, Argentina; emiliasoto@exactas.unca.edu.ar (M.E.S.A.); marioperea1964@yahoo.com (M.P.); 2 Instituto de Bioprospección y Fisiología Vegetal (IBIOFIV, UNT-CONICET), Facultad de Ciencias Naturales e IML, San Lorenzo 1469, San Miguel de Tucumán T4000CBG, Tucumán, Argentina; 3 Instituto de Morfología Vegetal, Fundación M. Lillo, Miguel Lillo 251, San Miguel de Tucumán T4000JFE, Tucumán, Argentina; airuiz@lillo.org.ar; 4 Cátedra de Anatomía Vegetal, Facultad de Ciencias Naturales e IML, Universidad Nacional de Tucumán (UNT), Miguel Lillo 205, San Miguel de Tucumán T4000JFE, Tucumán, Argentina

**Keywords:** cacti, anatomy, morphology, mycorrhizae, phenolic compounds, stomata

## Abstract

The Cactaceae family makes use of different strategies, both physiological and biochemical, for anatomical adjustments that allow them to grow and reproduce in arid environments. Morphological studies of *Gymnocalycium* have been scarce, and the anatomy and phytochemistry are still largely unknown. The aim of the present work was to analyze the structural, physiological, and biochemical features of *Gymnocalycium marianae* and *G. oenanthemum*, two endemic species of arid regions in Argentina. The anatomic structure, biomass, and photosynthetic pigments, as well as phenolic compound contents, were analyzed in the stem, spine, and root of both species. *G. marianae* showed stems with deeper substomatal chambers and a more developed photosynthetic tissue than *G. oenanthemum*. The spines of *G. oenanthemum* showed higher biomass, thicker epidermal and subepidermal cell walls, and a higher content of phenolic compounds than those of *G. marianae*. Ectomycorrhizae were observed for the first time in roots in both species. Roots of *G. marianae* showed high colonization, biomass, and content of phenolic compounds. Both species showed abundant mucilaginous fibers in the stem and root. Finally, these results show the strategies associated with the survival in xeric environments of two cacti species at risk of extinction. They could be useful for the development of ex situ conservation programs.

## 1. Introduction

The Cactaceae family is native to the American continent and comprises approximately 200 genera and 2500 species [1]. The genus *Gymnocalycium* has around 60 species and 7 subgenus; it is distributed in Paraguay, Brazil, Bolivia, Uruguay, and Argentina [2,3]. The province of Catamarca in Argentina has around 14 species of this genus; four of them are endemic, i.e., *Gymnocalycium baldianum* (Speg.) Speg. and *G. marianae* Perea; O. Ferrari, Las Peñas & R. Kiesling (subgenus *Gymnocalycium*); *G. stellatum* Speg. *occultum* Ssp. Fric ex H. Till & W. Till (subgenus *Trichomosemineum*); and *G. oenanthemum* Backeb. (subgenus *Microsemineum*). Each of these species has a limited distribution and inhabits specific climatic conditions, i.e., mean annual temperatures and precipitation that range between 9.5 and 19.7 °C and between 380 and 670 mm, respectively [4]. The species used in the present work, *G. oenanthemum* and *G. marianae,* grow in arid phytogeographic regions, which differ mainly in altitude and rainfall. These species are included in the red list of endangered species, in the categories “Endangered” (EN) and “Vulnerable” (VU), respectively [5]. The risk these populations run is closely related to the advance of the human population over the area, agricultural activities, and the commercialization of specimens extracted from their habitats due to the rising interest in the beauty of their flowers and shapes [2].

Most of the cacti growing in hostile environments, namely those with arid soils, high solar radiation, strong winds, or a high degree of salinity, among other factors, present adaptive strategies fixed during evolution that allow them to survive and reproduce [6]. The adaptive mechanisms are constitutive, so neither the expression nor the phenotypic manifestation of associated genes vary in different environmental conditions, while the acclimatization processes are induced according to environmental conditions. Different adaptations have been described in Cactaceae, i.e., crassulacean acid metabolism (CAM) photosynthetic pathways, leaves modified into spines, providing both resistance to water loss and extreme temperatures and defense against herbivores, synthesis of secondary metabolites, succulent stems with the presence of mucilage (polysaccharides), aimed at absorption, and storage of water [7]. During drought, the stem remains hydrated thanks to the supply of water from non-photosynthetic tissue made up of thin and flexible cell walls; in this way, the concentration of solutes by polymerization of carbohydrates can decrease [7]. Other strategies associated with the capture of resources, such as nutrients, minerals, and water, are symbioses with microorganisms, namely, bacteria and fungi. To date, only endomycorrhizal-type fungal associations have been reported in cacti [8,9].

Several histological studies on species of Cactaceae have been undertaken; however, research was mostly conducted on a particular organ for taxonomic or descriptive purposes [4,10,11,12,13,14,15].

We hypothesized that the *Gymnocalycium* species presents morphoanatomical and biochemical characteristics that are closely related to physiological processes and adaptive strategies in extreme arid environments.

The aim of the present work was to analyze the adaptative strategies of *G. marianae* and *G. oenanthemum*, two endemic cacti species, i.e., structural, physiological, and biochemical features, to arid regions in Argentina. Accordingly, ripe fruits of *G. marianae* and *G. oenanthemum* were collected from their natural habitats, and 3.5-year-old plants were obtained from the seeds for the different assays. In addition, the roots of plants obtained from the habitats of both species were collected for morphoanatomical analyses.

## 2. Results

Due to the scarce information on morphoanatomy and growth of *Gymnocalycium*, our studies aimed at making a histological description of the stem, spine, and root of both plant species in relation to biomass distribution, photosynthetic pigments, and phenolic compound content.

### 2.1. Plant Morphology

**Figure 1:**
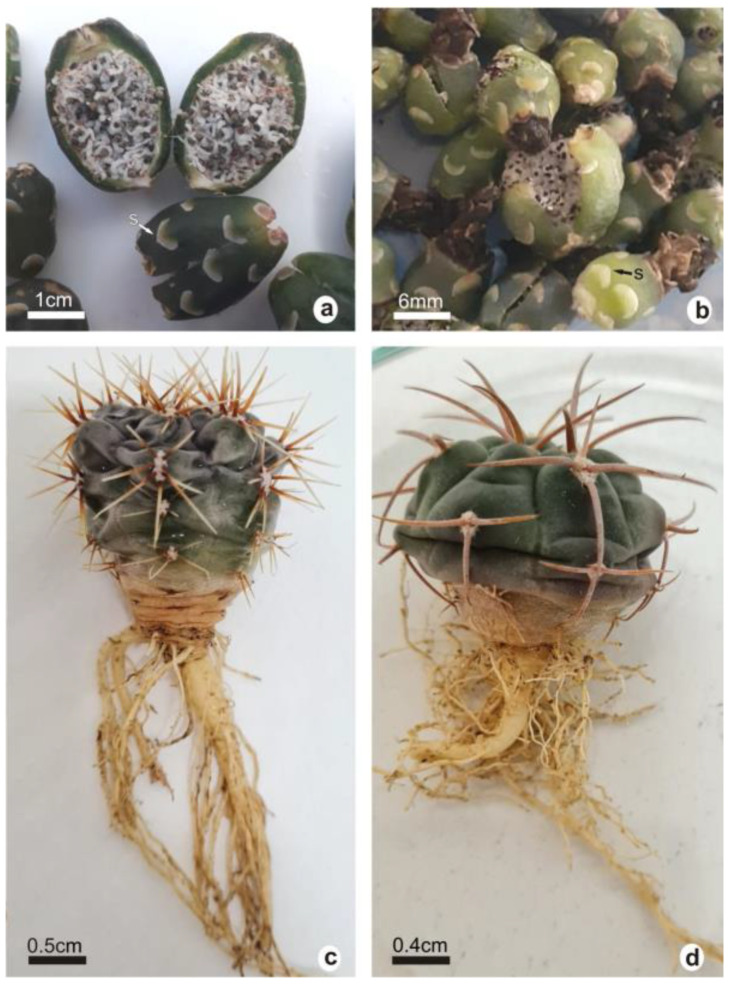
Plant material used for different studies. Fruits collected from plants in habitat (**a**,**b**); 3.5-year-old plants (**c**,**d**); *G. marianae* (**a**,**c**); *G. oenanthemum* (**b**,**d**). s, scale. The white and black bars represent the scale.

The fruits and 3.5-year-old plants of *G. marianae* and *G. oenanthemum* are shown in Figure 1. Fruits of both taxa were fleshy with rounded scales with a whitish-green edge on the surface, globose and dark green in *G. marianae* and oval and light green in *G. oenanthemum* (Figure 1a,b). *G. marianae* showed grayish-green stems with abundant fine, rigid, straight, dark spines at the base (Figure 1c). Instead, *G. oenanthemum* showed dark green stems with thick spines bent back towards the plant body (Figure 1d).

Stem length was 20% larger in *G. marianae* than in *G. oenanthemum*, whereas the stem diameter in the latter was 11% larger (Table 1). Both species showed an axonomorphic main root with lateral and adventitious roots, close to the neck, although thicker in *G. marianae* (Figure 1c,d). The root length did not present significant differences between both species (Table 1).

### 2.2. Photosynthetic Pigments

The photosynthetic pigment content of stems in both species is shown in Table 1. Chl *a* and Chl *b* content in *G. marianae* was higher than in *G. oenanthemum* by 47% and 31%, respectively. These different percentages result in differences in the Chl *a*/Chl *b* ratio between both plant species, where *G. marianae* showed a higher value, namely, close to 3, due to its higher Chl *a* content. In *G. marianae*, the carotenoid content was 1.6-fold higher than in *G. oenanthemum* (Table 1).

### 2.3. Biomass Distribution

The FW and DW in both species were higher in stems than in roots and spines (Table 2). Total DW in *G. marianae* was 20% higher than in *G. oenanthemum*. This latter species showed a high percentage of water in all its organs (Table 2). Regarding the dry weight distribution (DWD), although the stem showed a similar percentage, i.e., around 76%, in both species, it was substantially different in roots and spines (Table 2); *G. marianae* and *G. oenanthemum* showed 13.0% and 9.5% DWD in roots and 10.8% and 15.5% DWD in spines, respectively. The DW/FW ratio was higher in all organs of *G. marianae* compared to those of *G. oenanthemum*.

### 2.4. Ectomycorrhizae

**Figure 2:**
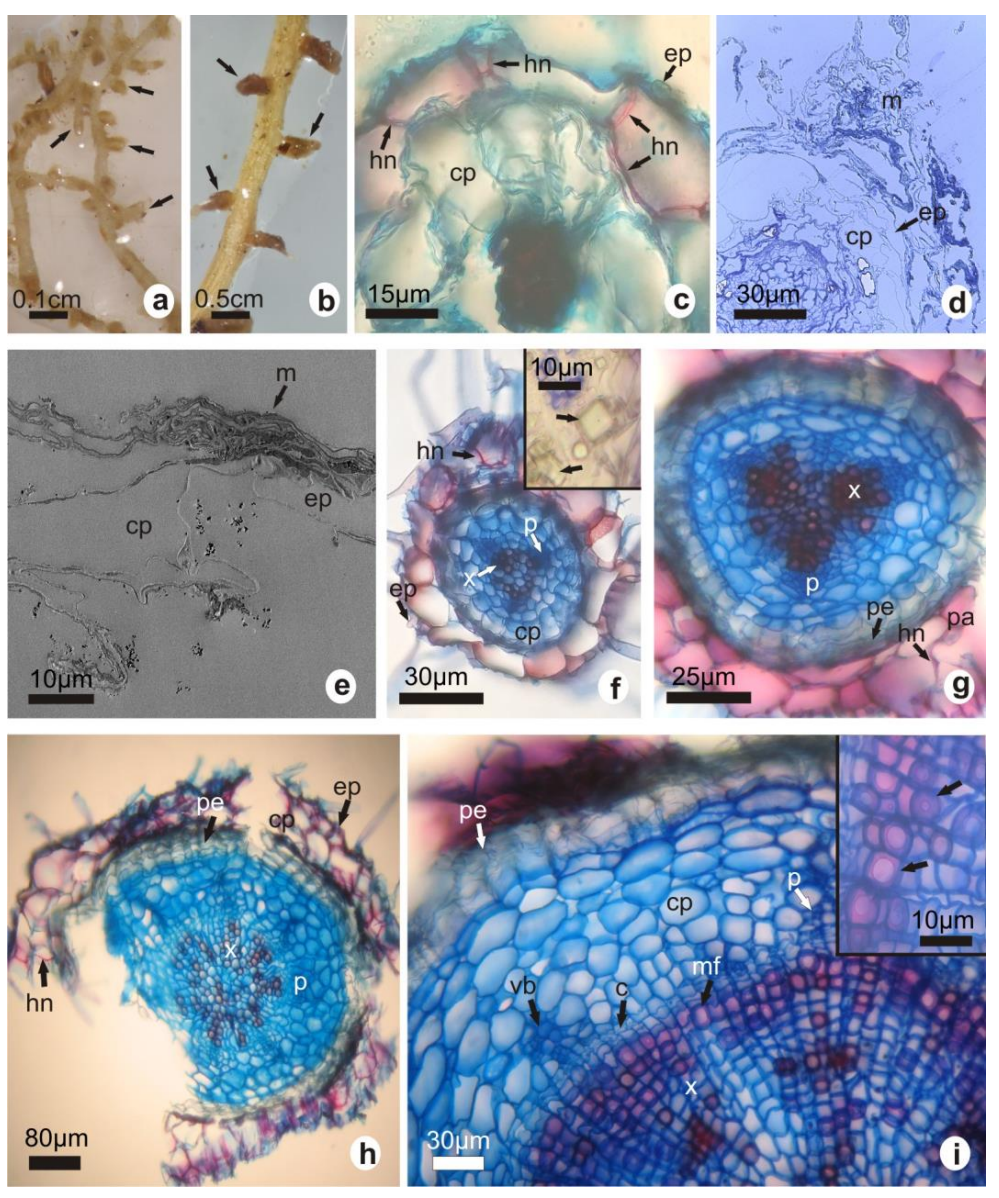
Ectomycorrhizae and root morphoanatomy. Roots with ectomycorrhizal colonization structures of simple morphological type (arrows) of greenhouse plants (**a**) and plants from their habitat (**b**). Primary radical structure (**c**,**f**). Prismatic calcium oxalate crystals in the cortex ((**f**), inset). Ectomycorrhizal intercellular hyphae (Harting net) in cortical parenchyma (red color) (**c**,**f**–**h**). Thin and ultrathin cross-sections denoting the fungus mantle (**d**,**e**). Early secondary root structures in different degrees of development (**g**,**h**). Secondary root structure with vascular bundles in the phloem and detail of mucilaginous fibers in the xylem (inset) (**i**). *G. marianae* (**a**–**e**,**g**–**i**); *G. oenanthemum* (**f**). c, cambium; cp, cortical parenchyma; ep, epidermis; hn, Hartig net; m, mantle; mf, mucilaginous fibers; p, phloem; pa, parenchyma; pe, periderm; vb, vascular bundles; x, xylem. The white and black bars represent the scale.

The lateral roots of 3.5-year-old plants obtained in a greenhouse exhibited noticeably short and swollen branches, corresponding to simple-type structures of ectomycorrhizae (Figure 2a). The number of structures observed was 19.28 ± 3.75 and 6.44 ± 2.23 in *G. marianae* and in *G. oenanthemum*, respectively (*p* < 0.0001). Both a thin mantle and a Hartig net corresponding to the intercellular ectomycorrhizae mycelium were detected in lateral root cross-sections in both species (Figure 2c–e). The important finding of ectomycorrhizae in plants grown in greenhouses led to the analysis of roots in both plant species in their habitats; the same symbiotic structures were found (Figure 2b).

The mantle was a 5–40 μm thick and irregular prosenchymatous structure. Mostly, the Hartig net penetrates into the first layer of the cortical parenchyma (Figure 2c–h, indicated with arrows).

### 2.5. Root Anatomy

The primary root structure showed an epidermis made up of one layer of cells, a parenchymal cortex made up of two layers of cells, and the endodermis. Prismatic calcium oxalate crystals were visualized in the cortex of *G. oenanthemum* only (Figure 2f, inset). The vascular cylinder showed a diarch stele and a multilayered pericycle (Figure 2f). The adventitious roots were structurally identical to the lateral roots, presenting triarch, tetrarch, and pentarch steles.

The secondary structure of roots showed different degrees of development. Early stages with primary tissues, i.e., epidermis, cortex with Hartig net, and endodermis, still attached to the developing periderm were more noticeable (Figure 2g,h). In the secondary structure, periderm, i.e., suber, phellogen, and phellodermis, cortical parenchyma with 6–9 cellular layers, phloem, and xylem, continuous due to cambium activity, and parenchymatical medulla were observed (Figure 2i). Vascular bundles in the phloem (Figure 2i) and mucilaginous fibers in xylem tissue (Figure 2i, inset) were noticed.

### 2.6. Stem Anatomy

**Figure 3:**
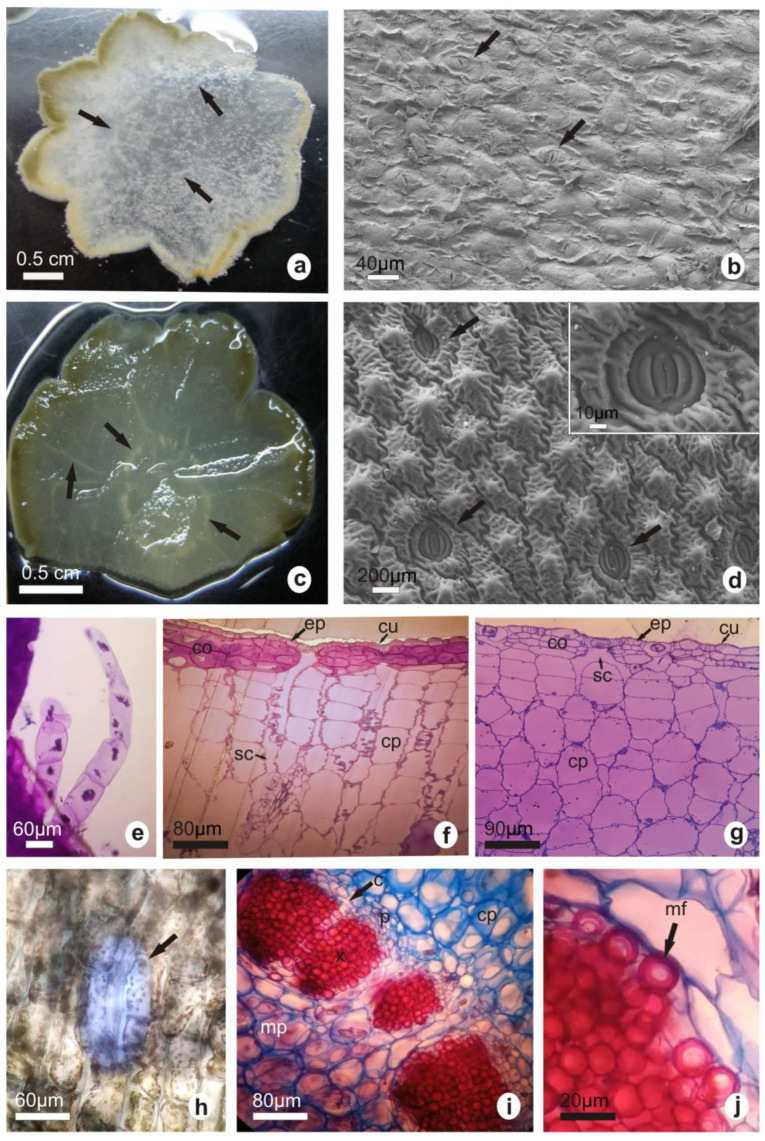
Stem anatomy. Cross-sections of stems visualized by stereomicroscopy denoting vascular bundles distributed as a ring or scattered in the cortex (**a**,**c**). Stem surface by SEM (arrow indicates stomata) (**b**,**d**). The inset in (**d**) shows a detail of stomata with two pairs of subsidiary cells. Optical microscopy of epidermal glandular trichomes (**e**). Cross-section of stem tissue visualized using light microscopy (**f**,**g**). Idioblasts containing mucilage (**h**). Cross-sections showing detail of vascular bundles (**i**,**j**). *G. marianae* (**a**,**b**,**e**,**f**,**h**); *G. oenanthemum* (**c**,**d**,**g**,**i**,**j**). c, cambium; co, collenchyma; cp, cortical parenchyma; cu, cuticle; ep, epidermis; p, phloem; mf, mucilaginous fibers; mp, medullary parenchyma; sc, substomatal chamber; x, xylem. The white and black bars represent the scale.

Stem cross-sections of both species showed round shapes; obtuse lobes with elongated centers and sharp vertices in *G. marianae* (Figure 3a) and rounded in *G. oenanthemum* (Figure 3c) could be seen. Vascular bundles, some scattered and others distributed as a ring, were found in the cortex (arrows in Figure 3a,c).

Square, as well as oblong, cells with sinuous walls were found in the epidermis, in paradermal view. The cuticle was smooth in *G. marianae* (Figure 3b) and strongly striated with papillae in *G. oenanthemum* (Figure 3d). Stomatal structure (paracytic-type) was similar in both species (Figure 3d, inset); however, size and density were higher in *G. marianae* than in *G. oenanthemum* (Table 1). Glandular trichomes with a multicellular uniseriate foot and unicellular head were observed in both taxa (Figure 3e).

Figure 3f,g shows stem cross-sections. Both species showed an epidermis consisting of one cell layer, with a thick and smooth cuticle in *G. marianae,* but a thin and striated one in *G. oenanthemum*. *G. marianae* showed greater thickness in the cell walls of the subepidermal collenchyma. Parenchyma with 4–5 and 5–7 layers of square or oblong cells with 20–23 and 4–5 elongated cell layers and 20–25 and 17–19 circular cell layers was found in *G. marianae* and *G. oenanthemum*, respectively. The deep substomatal chambers were observed, extending throughout 2–3 subepidermal lamellar collenchyma layers in both species, whereas parenchyma was observed in up to 5–6 layers in *G. marianae* and in 1–2 layers in *G. oenanthemum.*

Both elongated and round idioblasts containing mucilage in the outer area of the cortex (Figure 3h), alongside collateral vascular bundles distributed in a ring with cambium differentiation in some cases (Figure 3i), were found in both species. Also, dispersed bundles in the medulla (not shown) and in the cortex (Figure 3a,c), medullar parenchyma (Figure 3i), as well as xylem with numerous mucilaginous fibers, were found (Figure 3j).

### 2.7. Spine Anatomy

**Figure 4:**
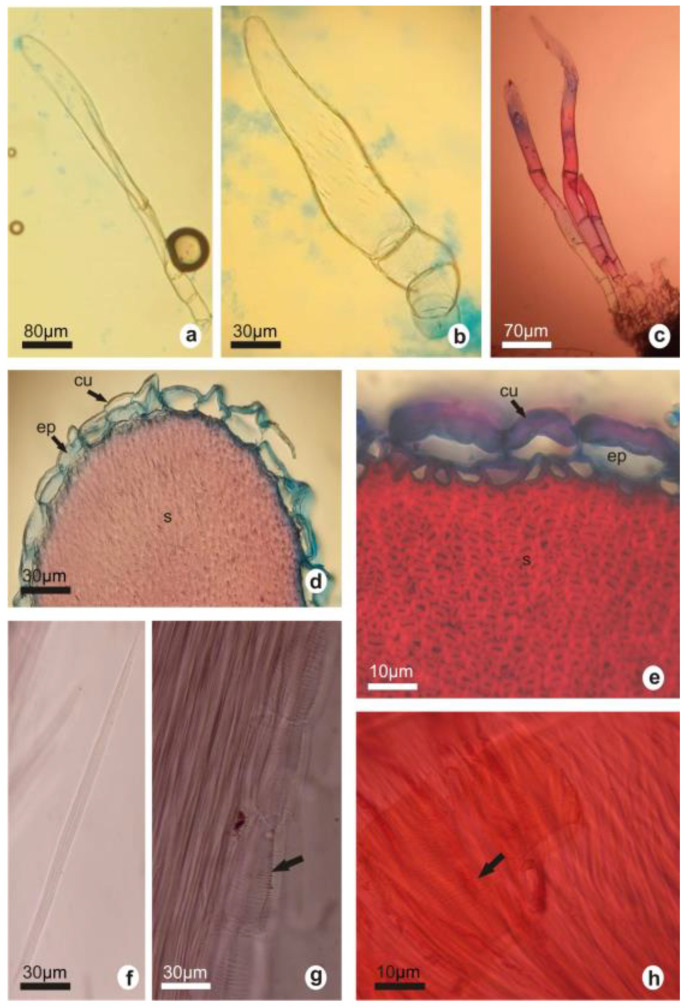
Spine anatomy by optical microscopy. Glandular trichomes (**a**–**c**). Spine cross-section (**d**,**e**). Fibers in dissociated samples (**f**). Longitudinal sections showing fibers and vessels with areolate pits (**g**) and helical thickening (**h**). *G. marianae* (**a**,**b**,**d**,**g**); *G. oenanthemum* (**c**,**e**,**f**,**h**). cu, cuticle; ep, epidermis; s, sclerenchyma. The white and black bars represent the scale.

Glandular trichomes with pluricellular uniseriate feet and unicellular heads could be seen at the spine bases of both species. Trichomes in *G. marianae* were of two types: the thin, elongated, smooth-cuticled ones (Figure 4a), and the thick and short ones with a striated cuticle (Figure 4b). *G. oenanthemum* showed thin and elongated trichome clusters with a smooth cuticle (Figure 4c).

In spine cross-sections, both species showed a circular shape with an epidermis made up of one cellular layer and a smooth cuticle. Papillose epidermises without and with a thickened outer periclinal wall were observed in *G. marianae* and *G. oenanthemum*, respectively (Figure 4d,e). The cortex was formed by numerous layers of sclerenchyma with or without lignification in the outermost layer (Figure 4e). The dissociation of the spine showed that the sclerenchymal tissue is formed by fibers (Figure 4f), macrosclerids, and segments of short vessels with areolate pits in *G. marianae* (Figure 4g) and helical thickening in *G. oenanthemum* (Figure 4h).

### 2.8. Phenolic Compounds

**Figure 5:**
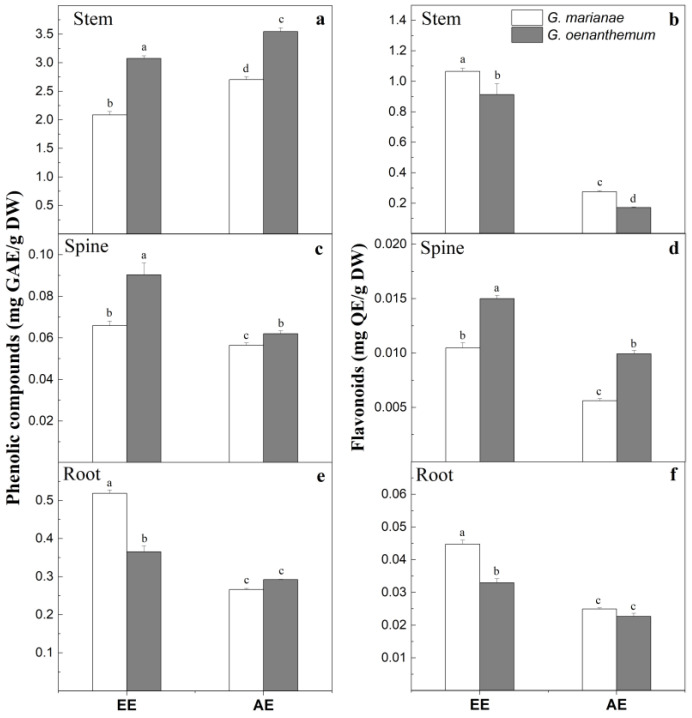
Phenolic compounds (**a**,**c**,**e**) and flavonoids (**b**,**d**,**f**) of ethanolic extracts (EE) and aqueous extracts (AE) from stems, spines, and roots of *G. oenanthemum* and *G. marianae*. Mean ± SD; same letter, not significantly different in each organ (Student test, *p* < 0.05). GAE: gallic acid equivalent; QE, quercetin equivalent; DW: dry weight.

Total phenol compound content was higher in stems than in roots and spines in both plant species; the content of these compounds was higher in ethanolic extracts than in aqueous extracts (Figure 5).

Stem phenolic compound contents in *G. oenanthemum* were 3.07 mg GAE/g DW (ethanolic extract) and 3.55 mg GAE/g DW (aqueous extract); in *G. marianae,* however, the phenolic content was lower than in *G. oenanthemum* (32 and 23%, respectively) (Figure 5a). The total phenolic compound content of ethanolic extracts from spines was 37% higher in *G. oenanthemum* compared to *G. marianae* (Figure 5c). The *G. marianae* root ethanolic extract showed a higher content of total phenolic compounds than the *G. oenanthemum* root extract by 42% (Figure 5e).

The flavonoid content was lower in the spines and roots than in the stem. However, the flavonoid content was higher in *G. marianae* stem than in *G. oenanthemum* stem (Figure 5b).

## 3. Discussion

The present work showed adaptive and acclimation strategies in *G. marianae* and *G. oenanthemum* in arid regions. The presence of ectomycorrhizae was demonstrated for both *Gymnocalycium* species, both in those grown in greenhouses and in those collected from their natural habitats. Ectomycorrhizae were reported in some members of the families Pinaceae, Fagaceae, Salicaceae, Betulaceae, Myrtaceae, and Fabaceae, but this is the first report in Cactaceae [7,16]. Up to date, only arbuscular mycorrhizal fungi associations have been found in species of Cactaceae of the genera *Opuntia, Trichocereus, Gymnocalycium,* and *Acanthocalycium* [9], and *Pachycereus, Machaerocereus*, and *Lemaireocereus* [8]. Ectomycorrhizae (basidiomycete and ascomycete) have evolved independently from saprotrophic ancestors and retained some of their functional genes, thus leading to differences in the mode and capacity of organic matter decay [17].

The association root-mycorrhizae is central for plant nutrition and water capture, as well as environmental stress responses in some plant species [18,19,20]. Thus, the ectomycorrhizal symbiosis detected in the species of *Gymnocalycium* could result in an adaptation mechanism for tolerance to xerophytic environments.

The fungal colonization was higher in *G. marianae* than in *G. oenanthemum*, a fact that can be linked to the higher root biomass and the higher phenolic compound content. The increase of these compounds was also observed in the interaction between silver birch and ectomycorrhizae [21]. The higher root biomass of *G. marianae* reveals a greater sink force of photosynthates, which could be a consequence of the symbiotic association. This, in turn, can be linked with the higher development of photosynthetic parenchyma in this plant, i.e., number of cell layers, higher density of stomata, and photosynthetic pigment content, in comparison to *G. oenanthemum.* High stomatal density in *G. marianae* was also reported by Perotti et al. [4]. Moreover, other differential traits of *G. marianae* have been detected in the stem: cuticle thickness, subepidermal collenchyma with thick cell walls, and major carotenoids and flavonoid content could protect the stem against winds, desiccation, and high solar radiation.

A very interesting finding in *G. marianae* was the presence of substomatal chambers of great depth, which extend to the fifth to sixth layer of parenchyma. This fact would correspond to a particular adaptive strategy for water conservation in xeric environments. It has been reported that substomatal chambers cross only up to the “hypodermis” in species of *Cipocereus* (Cactaceae) [12] and extend into the first layer of the cortical parenchyma in *Opuntia* species [22]. The control of guard cell opening and closing, as well as the depth of the substomatal chambers, is of great importance to the efficiency of water use. Guard cell openings in CAM plants appear to be unresponsive to blue light; instead, they present a strong sensitivity to the vapor pressure deficit (VPD) in the plant-atmosphere system [23]. In this sense, the participation of the very deep substomatal chambers of *G. marianae* could play a fundamental role in the regulation of VPD.

Water conservation is also associated with the presence of mucilage-containing idioblasts and the numerous mucilaginous fibers detected in both species. The function of mucilaginous fibers in the xylem tissue of stems and roots with secondary growth has not been thoroughly studied to date. Thus, they would participate in regulating the flow of water through the plant by changes in mucilage hydric potential and could absorb and retain water when the stomata are closed and release the water towards the stomata when they are open, i.e., during the night or by VPD regulation. Thus, one of the proposals of the present work is to consider mucilaginous fibers as adaptive structures required for the flow of water through xylem in CAM plants.

The *G. oenanthemum* spines showed major biomass, thicker cell walls, i.e., epidermis and subepidermis, and higher phenolic compound content than those of *G. marianae*, a fact that could be associated with defense against herbivores. Vessels with helical thickening were present in the sclerenchymatic tissue of *G. oenanthemum* spines. This could be related to the flexibility required during the formation of the curved spines. *G. marianae* showed vessels with areolate pits and straight spines. Helical thickenings present in tracheids are known to provide more organ flexibility compared to tracheids with areolate pits [24]. The helical thickening or areolate pits found in the vessels, characters associated with the morphology of the spines, could indicate that both species have different adaptations for defense against herbivores or other environmental factors.

The papillae on the *G. oenanthemum* stem surface, as well as the trichomes found between ribs and at the base of the spines in both species, could help retain and take atmospheric water inside the plant. Papillae are rare in cacti, except in species of *Pterocactus,* and they are considered an adaptation to arid environments [25,26].

## 4. Materials and Methods

### 4.1. Habitat Traits of Species under Study

**Figure 6:**
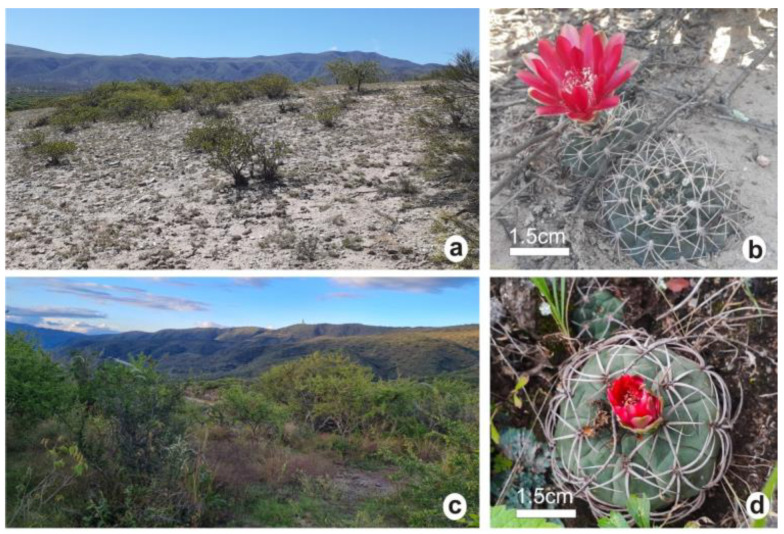
Habitat of species studied; photographs of the growth environment and flowering plants. *G. marianae* (**a**,**b**); *G. oenanthemum* (**c**,**d**). The white bars represent the scale.

*G. marianae* grows in Andalgalá, Catamarca, Argentina (27°40′ S, 66°01′ W), between 1600 and 1800 m.a.s.l. The plant population is located on the northern slope of Sierra de Ambato, on undulating hills exposed to both water and wind erosion. The soil is composed of coarse sediment and rocky outcrops of metamorphic origin. The mean annual precipitation is around 380 mm, with a marked dry season from May to September, with less than 10 mm per month. The rains are concentrated in the warm season, i.e., January to March. The mean annual temperature is around 9.5 °C. Frosts occur from April to October–November [27]. *G. marianae* grows in an ecotone of mount and prepuna with scarce vegetation. Other plants found in the region are *Prosopis nigra* (algarrobo negro) and, less frequently, *Condalia microphila* (piquillín pispa) and *Larrea cuneifolia* (jarilla macho) (Figure 6a,b). The *G. marianae* habitat region was inhabited by the Incas until the mid-16th century, as evidenced by archaeological pieces [28] found in the area.

*G. oenanthemum* grows in Ambato, San Fernando, and Capayán, three districts in Catamarca between 500 and 1000 m.a.s.l. The plant material was collected from the east slope of Sierra de Ambato, Ambato (28°16′ S, 65°52′ W). The soil is rocky and of metamorphic origin, with abundant superficial organic matter. The mean annual precipitation is about 670 mm, with rainy and dry periods from October to March and from April to September, respectively. The mean annual temperature is 15 °C, with frosts occurring from April to October [29]. The ecoregion corresponds to Chacoan grassland, with abundant herbaceous vegetation and stray cattle. The plants are either exposed to full sunlight or protected by shrubs, coarse grasses (*Stipa* sps.) and trees such as *Vachellia caven* (churqui) (Figure 6c,d).

### 4.2. Plant Material

Ripe fruits of 12 plants of both *G. marianae* and *G. oenanthemum* were collected from their natural habitats in Andalgalá and Ambato, respectively, during January and February 2017 (Figure 1a,b). The seeds were extracted from the fruits, washed, dried, and stored at room temperature. The seeds were germinated and grown for one year in plastic trays with commercial organic substrate “Bertinat” and kept under greenhouse conditions; temperatures in the greenhouse ranged between 10 and 35 °C and relative humidity between 40 and 60% in winter and summer. Then, the plants were transferred to a speeding tray containing peat for them to continue growing for up to 3.5 years. Then, 25 plants of each species were randomly collected, and the roots were carefully washed. These plants, which were used for all the assays, were freeze-dried and powdered for phytochemical analysis. The fresh plants were used for morphological characterization.

In order to examine whether the plants grown in the habitat present mycorrhizal symbioses, an independent assay was carried out. For this purpose, roots were collected from 5 individuals of both *G. marianae* and *G. oenanthemum* coming from Andalgalá and Ambato, respectively. FAA (formaldehyde: acetic acid: 50% ethanol, 5:5:90, v:v:v) was used to fix the roots until the assays were carried out.

### 4.3. Morphological Characterization, Photosynthetic Pigments, and Biomass

The diameter and height of the stem, as well as the root length, were recorded using a digital caliper.

Chlorophyll and carotenoids were extracted from 30 mg of fresh stem with 2 mL of dimethyl sulfoxide for 12 h at 45 °C. The chlorophyll *a* (Chl *a*), chlorophyll *b* (Chl *b*), and carotenoid contents were calculated from absorbances at 665, 649, and 480 nm according to Wellburn [30] and expressed as µg g^−1^ FW.

The root, stem, and spine were freeze-dried, and both the fresh and dry weights (FW and DW) were determined. DW/FW ratio, water content, and DW distribution percentage (DWD) [(∑ DW root, stem, and spine/DW each organ) × 100] were calculated.

### 4.4. Ectomycorrhizae Morphology

The morphological types of ectomycorrhizal roots were determined according to Agerer [31] from habitat and greenhouse grown plants. The number of ectomycorrhizal branches in lateral roots was evaluated through observations using a stereomicroscope (Olympus Co, Olympus SZX7, Tokyo, Japan).

### 4.5. Anatomical Analysis

The anatomical analysis was performed on fresh plant material, namely roots, stems, and spines, fixed in FAA. Free-hand cross-sections were made from the equatorial zone of the stem and spine and serial sections from all the radical systems. To characterize the anatomical structure, a successive double astra blue-safranin stain was used and subsequently mounted in water–glycerine (1:1, *v*/*v*) [32,33]. Spine dissociation was performed using the Schultze technique [32].

Stoma types were determined by using the Dilcher classification [34]. The length, width, and density of stomata were quantified.

A histochemical test was performed for the detection of mucilage (cresyl blue 1%) [32].

The observations were made by using a stereomicroscope (Olympus Co, Olympus SZX7, Tokyo, Japan) and a light microscope (Carl Zeiss, Axiostar Plus, Göttingen, Germany) coupled to a digital camera (Canon A620, Power Shot 7.1 MP).

Root and stem sections were fixed in Karnovsky’s solution (4% formaldehyde, 5% glutaraldehyde, and 0.1 mol·L^−1^ sodium phosphate buffer, pH 7.4) [35] and post-fixed with a 1/1 solution of sodium phosphate buffer with 2% osmium tetroxide. After incubation at 4 °C overnight, samples were washed, embedded in Spurr resin, and cut with an ultra-microtome to obtain thin (3 µm) and ultrathin (100 nm) sections. Thin sections were stained with 0.05% toluidine blue [36] and observed by using an optical microscope (Carl Zeiss, Axiostar Plus, Göttingen, Germany) coupled to a digital camera (Canon A620, Power Shot 7.1 MP).

The slides analyzed were deposited at the Institute of Plant Morphology of Miguel Lillo Foundation, Tucumán, Argentina.

### 4.6. Transmission (TEM) and Scanning (SEM) Electron Microscopy

The ultrathin sections of the root were mounted on copper grids and contrasted with uranyl acetate and lead citrate [37] for observation with a transmission electron microscope (Carl Zeiss, EM109, NTS GmbH, Oberkochen, Germany).

Stem surface micromorphology was analyzed. The fragments fixed in Karnovsky’s solution were dehydrated with alcohol and acetone. Next, the preparations were critical-point dried in liquid CO_2_, sputter-coated with gold-palladium, and observed with SEM (Zeiss Supra 55VP, Carl Zeiss, Oberkochen, Germany). SEM micrographs were taken with a digital camera (Olympus SP350, 8.0 MP, Tokyo, Japan). SEM and TEM studies were performed at Centro Integral de Microscopía Electrónica (CIME-CONICET, Tucumán, Argentina).

### 4.7. Soluble Phenolic Compound Determination

The samples were freeze-dried and ground. The soluble phenolic compounds were extracted in ethanol 80° and water for 1 h at room temperature by using different DW/solvent ratios, as follows: 1:4 for stem and 1:12 for spine and root in ethanolic extracts, and 1:18 for stem, 1:12 for spine, and 1:13 for root in aqueous extracts. Total phenol content was determined according to Singleton et al. [38]. Results were expressed as mg gallic acid equivalents/g DW (mg GAE/g DW). Total flavonoids were estimated by Woisky and Salatino [39]. Results were expressed as mg of quercetin equivalents/g DW (mg QE/g DW).

### 4.8. Statistical Analysis

Statistical differences in morphological features (*n* = 20), biomass (*n* = 20), pigment photosynthetic content (*n* = 5), and phenolic content (*n* = 5) were determined by analysis of variance (ANOVA) followed by Tukey’s test (*p* < 0.01). The anatomical analyses were performed using five plants from each species. The stomata density was quantified for *n* = 10 fields at × 40 objective lenses, with three replications for each one. Both the length and width of the stomata were evaluated (*n* = 50). The density and size of stomata and the number of ectomycorrhizal structures were expressed as mean ± standard deviation and compared by using Student’s *t* tests.

## 5. Conclusions

The present study shows a detailed anatomical description strongly correlated with physiological aspects of two *Gymnocalycium* species. Different adaptive strategies are found in *Gymnocalycium* species that allow them to survive in xeric environments. To our knowledge, this is the first report on the presence of ectomycorrhiza in Cactaceae. It is essential to further the study of adaptation mechanisms of plants resistant to extreme environments, taking into account the global changes of the planet and the risk of extinction of plant species due to human activities.

## Figures and Tables

**Table 1:** Morphometric analysis and photosynthetic pigments from *G. marianae* and *G. oenanthemum*.

Organs and Stomata	*G. marianae*	*G. oenanthemum*
Stem diameter (mm)	28.30 ± 1.90 ^b^	31.48 ± 2.67 ^a^
Stem length (mm)	32.87 ± 5.77 ^a^	27.30 ± 4.82 ^b^
Root length (mm)	57.74 ± 12.79 ^a^	64.26 ± 14.89 ^a^
Stoma length (µm)	101.2 ± 5.0 ^a^	88.4 ± 5.3 ^b^
Stoma width (µm)	79.8 ± 3.5 ^a^	63.7 ± 4.4 ^b^
Stomatal density (mm^2^)	40.4 ± 2.7 ^a^	19.6 ± 1.5 ^b^
Photosynthetic pigments (µg g^−1^ FW)		
Chlorophyll *a*	405.35 ± 3.44 ^a^	275.98 ± 5.75 ^b^
Chlorophyll *b*	140.26 ± 6.20 ^a^	107.37 ± 18.51 ^b^
Chl *a*/Chl *b*	2.89 ± 0.19 ^a^	2.57 ± 0.15 ^b^
Carotenoids	73.25 ± 1.86 ^a^	46.54 ± 1.94 ^b^

Values are mean ± SD; different letters in the same arrow indicate significant differences by Student’s *t* test, *p* < 0.05.

**Table 2:** Biomass and water content from *G. marianae* and *G. oenanthemum*.

Parameters	*G. marianae*			*G. oenanthemum*		
	Stem	Spine	Root	Stem	Spine	Root
FW (g)	8.81 ± 1.78 ^a^	0.16 ± 0.06 ^a^	0.85 ± 0.30 ^a^	9.74 ± 2.02 ^a^	0.20 ± 0.08 ^a^	0.64 ± 0.21 ^b^
DW (g)	1.06 ± 0.20 ^a^	0.15 ± 0.05 ^a^	0.18 ± 0.06 ^a^	0.87 ± 0.15 ^b^	0.18 ± 0.07 ^a^	0.11 ± 0.03 ^b^
DWD (%)	76.26 ± 2.40 ^a^	10.79 ± 0.50 ^b^	12.95 ± 1.20 ^a^	75.00 ± 1.98 ^a^	15.52 ± 0.26 ^a^	9.48 ± 1.32 ^b^
DW/FW	0.12 ± 0.01 ^a^	0.92 ± 0.02 ^a^	0.21 ± 0.03 ^a^	0.09 ± 0.01 ^b^	0.88 ± 0.03 ^b^	0.18 ± 0.02 ^b^
Water content (%)	87.72 ± 1.91 ^b^	8.36 ± 3.05 ^b^	78.89 ± 2.70 ^b^	90.92 ± 1.62 ^a^	11.76 ± 3.11 ^a^	81.64 ± 2.96 ^a^

Values are mean ± SD; the same letter in each column indicates they are not significantly different in the same organ of different species (Student’s *t* test, *p* < 0.05). FW: fresh weight; DW: dry weight; DWD: dry weight distribution.

## Data Availability

All data are available in the present work.
